# Biomechanical Study and Analysis for Cardiovascular/Skeletal Materials and Devices

**DOI:** 10.3390/jfb14080398

**Published:** 2023-07-26

**Authors:** Aike Qiao, Tianming Du, Haisheng Yang, Yongliang Mu

**Affiliations:** 1 Faculty of Environment and Life, Beijing University of Technology, Beijing 100124, China; dutianming@bjut.edu.cn (T.D.); haisheng.yang@bjut.edu.cn (H.Y.); 2 School of Metallurgy, Northeastern University, Shenyang 110819, China; muyl@smm.neu.edu.cn

The Special Issue entitled “Biomechanical Study and Analysis for Cardiovascular/Skeletal Materials and Devices” addresses biological functional materials and devices relevant to cardiovascular diseases and orthopedic conditions. We are grateful for the opportunity provided by the *Journal of Functional Biomaterials* and the strong support of the researchers involved.

This Special Issue comprises a total of 22 articles, covering a wide range of interventional medical devices in the cardiovascular and orthopedic fields. The research presented in this Special Issue covers various applications of polymer biomaterials, metal biomaterials, organic biomaterials, and composite biomimetic materials [1,2,3,4,5,6]. The primary focus of this Special Issue is to investigate the biomechanical properties of cardiovascular/skeletal materials and devices, reflecting current research interests. In the subsequent sections, we provide a concise overview of the key aspects and challenges in the study of commonly used biological functional materials in cardiovascular and orthopedic fields. We also discuss future research trends, drawing insights from the articles featured in this Special Issue.

**1.**  **Why Study the Biomechanical Properties of Biomaterials for Cardiovascular/Skeletal Applications?**

Cardiovascular and orthopedic diseases are two global epidemic diseases with far-reaching medical and socio-economic consequences [7]. Understanding the mechanical characteristics of arterial walls, blood flow, and valves holds great importance for the diagnosis, management, and treatment of cardiovascular diseases [8,9,10]. At the same time, bone tissue serves as a crucial weight-bearing structure within the body [11]. Consequently, the mechanical properties of implant materials and devices play a pivotal role in addressing cardiovascular and orthopedic diseases.

**2.**  **Research Progress of “Biomechanical Study and Analysis for Cardiovascular/Skeletal Materials and Devices”**
  (1) **A new phenomenon of interstitial fluid (ISF) microflow in perivascular and adventitial spaces around neurovascular bundles was reported.** Within this Special Issue, Kong et al. presented novel observations regarding the microflow of interstitial fluid (ISF) within the perivascular and adventitial clearances (PAC) surrounding neurovascular bundles [12]. This study not only enhances our understanding of ISF circulation throughout the body but also provides insights into the fundamental architecture of PAC. It helps to lay the foundation for the kinematics and dynamics of the ISF flow along the PAC around neurovascular bundles. Consequently, it establishes a basis for investigating the kinematics and dynamics of ISF flow along the PAC surrounding neurovascular bundles.
  (2) **Matching of mechanical properties and degradation performance of metal biomaterials is currently a focus of attention in both orthopedic and cardiovascular materials** [13]. In this Special Issue, Zhang et al. conducted numerical simulations to investigate material degradation [14]. Their study revealed that the suggested non-uniform degradation model, incorporating multiple factors for biodegradable endovascular stents, exhibited distinct phenomena when compared to commonly employed models. Furthermore, the numerical simulation results were found to align more closely with real-world degradation scenarios. In addition, various biodegradable porous materials were developed and demonstrated favorable compatibility between degradation and mechanical properties. For instance, biodegradable porous zinc stents and high-strength porous hydrogels showed promising biocompatibility for bone tissue engineering [2,3].
  (3) **Improving biomaterials through biomimetic mineralization.** The composite properties of cardiovascular and skeletal systems play a vital role in their remarkable functionality as human tissues. In recent studies, there has been considerable interest in the investigation of biomimetic materials. Du et al. highlighted the significance of collagen mineralization research, which not only provides insights into the formation mechanisms of physiological tissues in humans but also holds promise for the development of more suitable biological functional materials for treating orthopedic diseases [11,15].
  (4) **Advanced imaging, detection equipment, and computer technology have also greatly promoted the development of this field** [16]. A balloon dilatation catheter plays a critical role in percutaneous transluminal angioplasty procedures. In this Special Issue, Li et al. aimed to enhance our understanding of the underlying patterns by employing a highly realistic simulation method for balloon folding. They compared the trackability of balloons constructed from different materials, seeking to provide more effective insights [1]. This simulation-based approach allows for the evaluation of balloon performance when navigating curved paths, offering more precise and detailed data feedback compared to traditional benchtop experiments. Additionally, Lv et al. conducted a meticulous quantification of coronary artery plaque morphology and predicted cap thickness and stress/strain index. They employed a combination of IVUS, OCT data, biomechanical models, and machine-learning techniques for accurate assessments [17]. Furthermore, Huang et al. developed an automatic multilayer segmentation and repair method to extract multilayer vessel geometries from OCT images, facilitating the construction of biomechanical models [18]. The proposed segmentation technique holds significant potential for wide-ranging applications in vulnerable plaque research.
**3.**  **Summary**

Through curating this Special Issue, our intention is to enhance researchers’ comprehension of the fundamental issues and challenges prevalent in current investigations concerning cardiovascular/skeletal materials and devices. Furthermore, we aim to offer theoretical foundations that contribute to resolving clinical problems associated with cardiovascular and skeletal diseases. In future endeavors, it is imperative to allocate more extensive attention to interdisciplinary investigations encompassing bone–vascular cross-talk, both at fundamental and clinical levels. Additionally, we emphasize the importance of translating existing research studies into practical clinical applications, thereby effectively addressing the pressing health concerns affecting humanity.

## Data Availability

The data presented in this paper are available on request from the corresponding author.
